# Fabrication of Continuous Microfibers Containing Magnetic Nanoparticles by a Facile Magneto-Mechanical Drawing

**DOI:** 10.1186/s11671-016-1646-8

**Published:** 2016-09-23

**Authors:** Jin-Tao Li, Xian-Sheng Jia, Gui-Feng Yu, Xu Yan, Xiao-Xiao He, Miao Yu, Mao-Gang Gong, Xin Ning, Yun-Ze Long

**Affiliations:** 1 Collaborative Innovation Center for Nanomaterials & Optoelectronic Devices, College of Physics, Qingdao University, Qingdao, 266071 China; 2 Industrial Research Institute of Nonwovens & Technical Textiles, Qingdao University, Qingdao, 266071 China; 3 Department of Mechanical Engineering, Columbia University, New York, NY 10027 USA; 4 College of Textiles & Clothing, Qingdao University, Qingdao, 266071 China

**Keywords:** Fibers, Magnetic force, Mechanical force, Polymeric composites

## Abstract

A facile method termed magneto-mechanical drawing is used to produce polymer composite microfibers. Compared with electrospinning and other fiber spinning methods, magneto-mechanical drawing uses magnetic force generated by a permanent magnet to draw droplets of polymer/magnetic nanoparticle suspensions, leading to fabrication of composite microfibers. In addition, because of the rotating collector, it is easy to control the fiber assembly such as fibrous array in parallel or crossed fibrous structure. The general applicability of this method has also been proved by spinning different polymers and magnetic nanoparticles. The resultant fibers exhibit good superparamagnetic behavior at room temperature and ultrahigh stretchability (~443.8 %). The results indicate that magneto-mechanical drawing is a promising technique to fabricate magnetic and stretchable microfibers and devices.

**Electronic supplementary material:**

The online version of this article (doi:10.1186/s11671-016-1646-8) contains supplementary material, which is available to authorized users.

## Background

Micro-/nanofibers have exhibited a lot of outstanding performance due to a very high specific surface-to-mass ratio and attracted much attention of various applications in the past two decades, such as filtration [1–3], biomedical science [4, 5], high-performance textile [6, 7], and catalysis [8]. By now, different methods have been developed to generate micro-/nanofibers, such as melt-blowing [9, 10], electrospinning [11–13], and jet spinning [14, 15]. Melt-blowing is capable of producing high yield microfibers with an average diameter within several microns. Electrospinning is one of the most versatile and efficient methods and has been explored extensively recently in the laboratory and applied to industry [16]. The electrospinning process can be simply summarized as the high voltage applies to the polymer solution, causing the solution electrification and leading to the fabrication of fibers. The fibers were stretched in the air with the solvent evaporated and finally collected on the collection device in the form of non-woven [17]. Each of these methods has their advantages; however, each of them has some deficiencies. For instance, the distribution of melt-blown fiber diameters is uneven [10]. The electric force generated by high voltage is needed to the formation of Taylor cone in electrospinning. The high voltage ranging from several kilovolts to tens of kilovolts may bring potential danger if improperly operated [18].

For the aim of increasing production, optimizing spinning process, and getting better micro-/nanofibers, the magnetic field has already been introduced into the spinning. For example, Wu et al. controlled stability of the electrospun fiber by applying a magnetic field in the electrospinning process [19]. Yang et al. also obtained aligned fibrous arrays and multilayer grids by appending the magnetic field generated by two parallel-positioned permanent magnets above the collection device [20]. Similar to Yang et al., Liu et al. introduced two bar magnets to the conventional configuration at the collector region, and electrospinning of aligned straight and wave polymeric nanofibers was achievable [21]. In these approaches, the magnetic field affects the movements of charged jet to get fibers with different arrangements and to control instability in electrospinning. However, the high voltage was still applied while the magnetic field just played an auxiliary role.

In this study, we use a facile method termed magneto-mechanical drawing to prepare microfibers. In this method, we regard the magnetic field as the main part of the spinning. Spinning solution droplet doped with magnetic nanoparticles was stretched under the magnetic force generated by a revolving permanent magnet to form a bridge and finally translated into continuous fibers. Magneto-mechanical drawing possesses lots of advantages compared with other common spinning methods. The equipment of magneto-mechanical drawing is low cost, safe, simple, and convenient without high voltage or high temperature. The resultant continuous fibers are aligned in regular order and can be easily transferred onto other substrates, like glass, silicon wafer, and plastic sheet. The resultant fibers that contain magnetic nanoparticles show excellent superparamagnetic behavior and ultrahigh stretchability and may have potential application in magnetic sensor or targeted drug delivery.

## Methods

### Materials

Poly(vinylidene fluoride) (PVDF; *M*_*w*_ ~ 550,000) and polymethyl methacrylate (PMMA; *M*_*w*_ ~ 350,000) were purchased from Aladdin and Sigma-Aldrich, respectively. Solvents we used include *N*, *N*-dimethylformamide (DMF; HCON(CH_3_)_2_) and acetone (CH_3_COCH_3_). Three kinds of magnetic nanoparticles (γ-Fe_2_O_3_, Fe_3_O_4_, and NiO) with average diameters of 20 nm were supplied by Aladdin.

### Preparation of Magneto-Mechanical Drawing Solution

The viscosity of solution and existence of magnetic nanoparticles are the decisive factors for the success of spinning. In this study, different concentrations of polymer and nanoparticles were studied to obtain suitable spinning solutions. It is found that the solution containing 20–25 wt.% PVDF and 2.0 ± 0.5 wt.% magnetic nanoparticles was a suitable proportion. When the concentration of magnetic nanoparticles was less than 2.0 ± 0.5 wt.%, magnetic force is not strong enough to attract the spinning droplet under higher rotating speed. On the other hand, fiber diameter will become larger when the concentration of magnetic nanoparticles was more than 2.0 ± 0.5 wt.%. Table 1 shows different concentrations of PVDF solutions, their viscosities, and the spinning results. In order to prepare the magneto-mechanical drawing solution with PVDF concentration of 22 wt.%, 2.2 g PVDF was dissolved into 7.8 g mixed solution of DMF and acetone (50/50, *w*/*w*) with magnetic stirring at 40 °C for 2.5 h to form the precursor solution. Then 0.2 g γ-Fe_2_O_3_ nanoparticles were added into the prepared solution. After 10-min vibration under ultrasonic accompanied by mechanical stirring, the final solution was stirred for another 10 min to ensure a better dispersion of γ-Fe_2_O_3_ nanoparticles in the solution. The Erlenmeyer flask, as the reaction vessel, was enclosed by sealing film because of the volatility of acetone. Meanwhile, γ-Fe_2_O_3_ can be replaced by other magnetic nanoparticles. Additional file 1: Figure S1 shows the viscosity versus shear rate curves for different PVDF/magnetic nanoparticle solutions.

**Table 1:** Different concentrations of PVDF spinning solutions, their viscosity for spinning, and their spinning results

PVDF concentration (wt.%)	Solution viscosity	Result of spinning
<20	Low	Difficult to get fibers
20–25	Modest	Suitable for spinning, good fibers
>25	High	Difficult to spin

### Magneto-Mechanical Drawing Set-up

Magneto-mechanical drawing set-up requires no high voltage or other harsh terms, it is simpler, easier to assemble, and safer compared with common spinning methods like electrospinning, melt-blowing. It mainly consists of three parts, including feeding device (a syringe pump (Longer Pump LSPO1-1A) and a syringe for solution), collection device (a stage (*r* = 4.5 cm) on which equipped with a permanent magnet (1 cm × 1 cm × 2.2 cm) and several pillars (*h* = 2.5 cm)) and a rotary motor. The rotary motor is connected to the stage and used to control the rotating speed of the stage. The magnet and pillars are vertically glued onto the stage. The pinhead is placed perpendicular to the direction of the pillar. The distance between them can adjust to several millimeters.

### Magneto-Mechanical Drawing Process

**Fig. 1:**
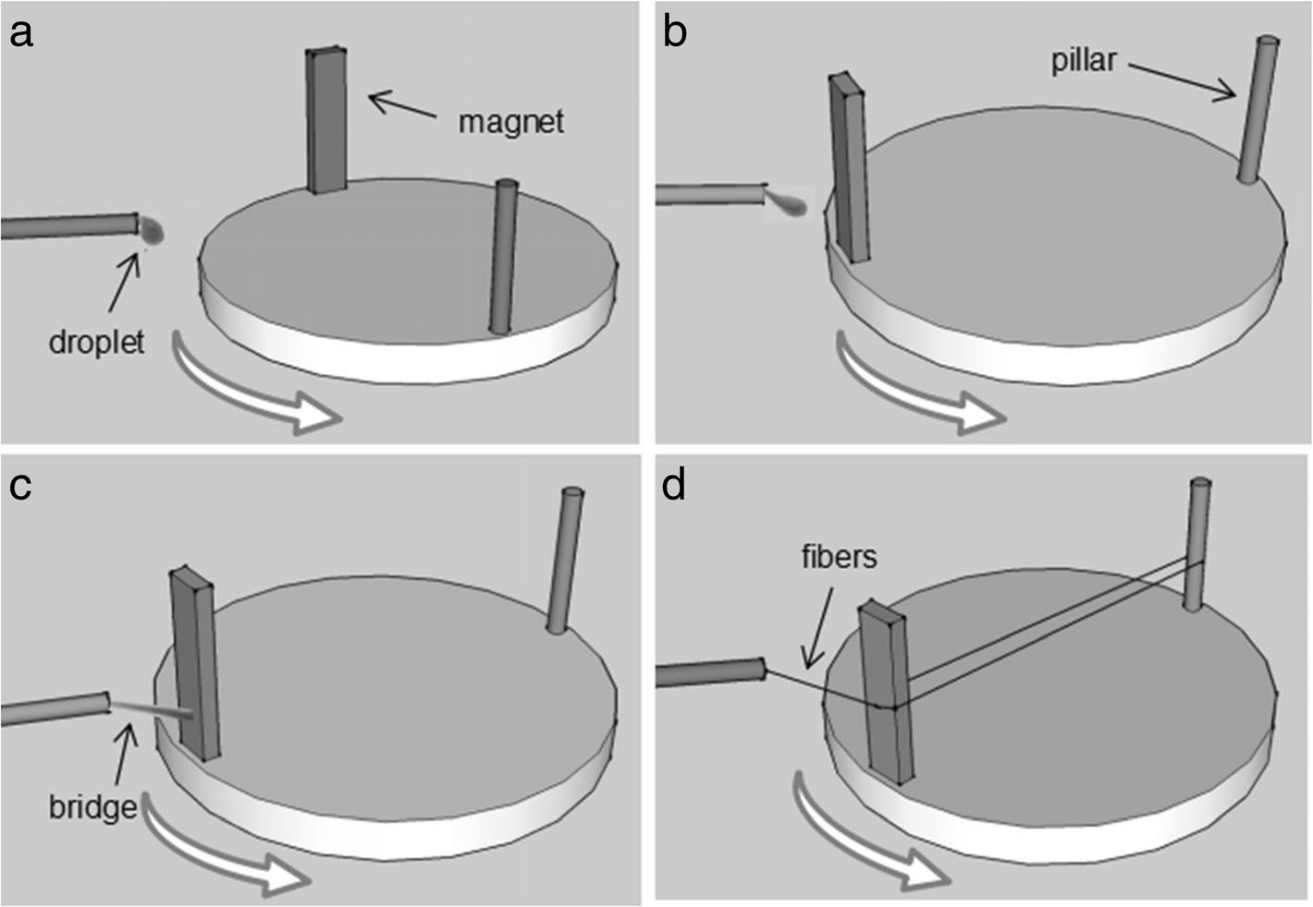
A schematic diagram of magneto-mechanical drawing process. **a** Magneto-mechanical drawing solution forms droplet at the pinpoint. **b** The magnet attracts droplet and **c** forms a bridge. **d** The bridge stretches rapidly and forms the fibers

**Fig. 2:**
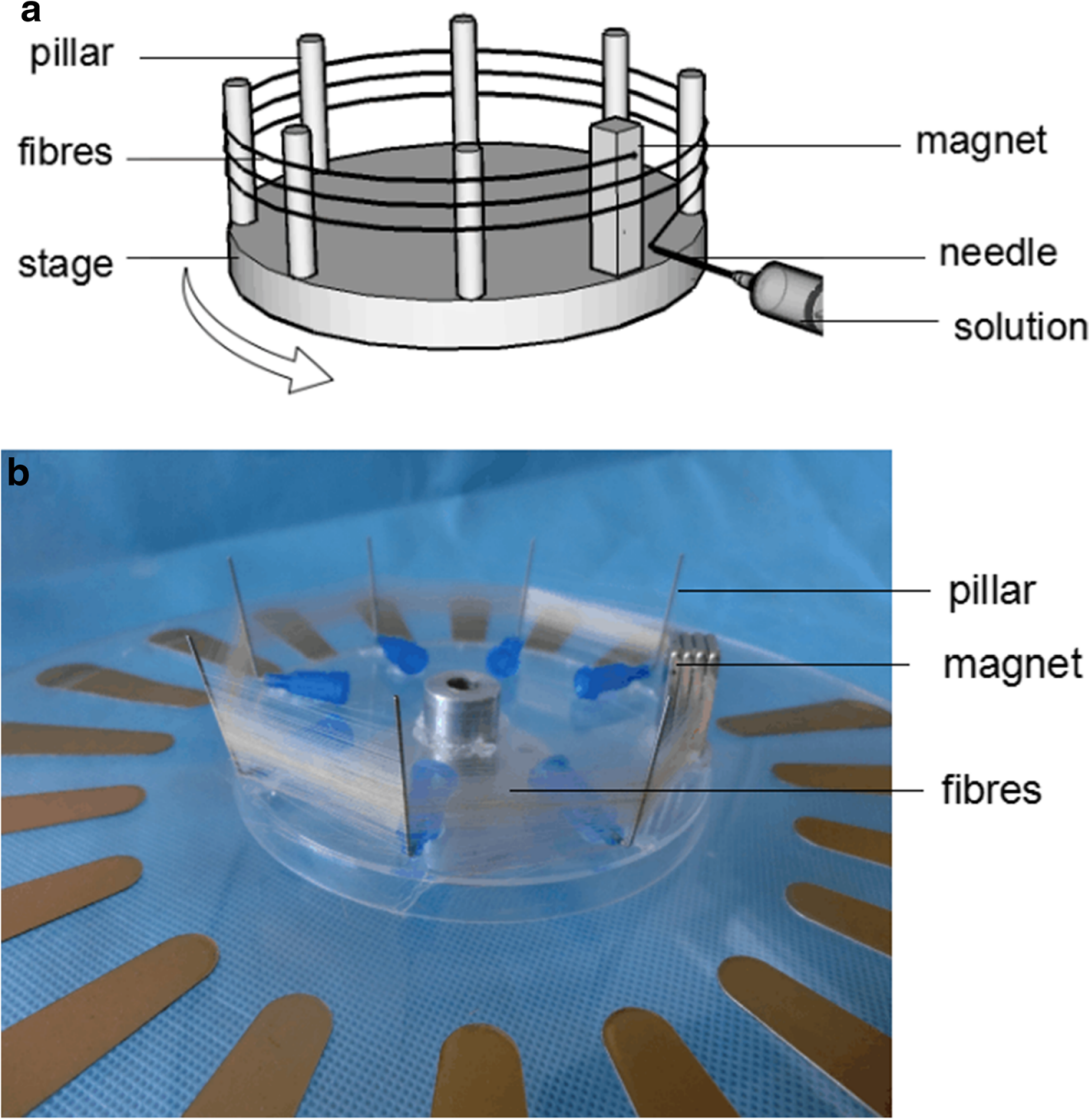
**a** Schematic diagram and **b** photograph of magneto-mechanical drawing collector with fibers

Generally speaking, magneto-mechanical drawing uses the magnetic force generated by a revolving permanent magnet to draw droplets of polymer suspensions with magnetic particles, leading to fabrication of microfibers. In a typical procedure, magneto-mechanical drawing solution is pushed out of the needle by the syringe pump, forming droplet at the pinpoint because of the surface tension (Fig. 1a). The stage with magnet starts to rotate at the same time. The magnet attracts droplet to form a bridge when it approaches to the needle tip (Fig. 1b, c). As the stage keeps rotating, the bridge stretches rapidly, forming the fiber (Fig. 1d). Fiber diameter decreases with its elongation. And fiber is drawn continually with the solvent evaporated. Once the fiber breaks, the droplet could be attracted to the magnet again. This process ensures the continuity of spinning. As shown in Fig. 2a, b, fibers are twining around the pillars and magnet to form a fibrous array in parallel. Besides parallel fibers, fibers with vertical cross structure can also be prepared by changing a removable box frame-collector.

### Characterization

The resultant composite fibers were characterized by a scanning electron microscope (SEM; JEOL, JSM-7500F), a transmission electron microscope (TEM; JEOL, JEM-2100F), and a Fourier transform infrared (FTIR) spectrometer (Thermo Scientific Nicolet iN10). The average diameter of the fibers was measured by using a SEM image analysis software (Smile View). The viscosities of different spinning solutions were measured by a rheometer (Physica MCR 301). The magnetic properties of the fibers were measured using a vibrating sample magnetometer (VSM) of physical properties measurement system (PPMS) of quantum design) by sweeping the external field from −25,000 to 25,000 Oe at 300 K. The stress-strain characteristic curve of fiber bundle (fiber number ~100, average fiber bundle diameter ~10 μm) was obtained by a dynamic mechanical analyzer (Q-800, TA Scientific).

## Results and Discussions

### Morphology and Structure of PVDF/γ-Fe_2_O_3_ Fibers

**Fig. 3:**
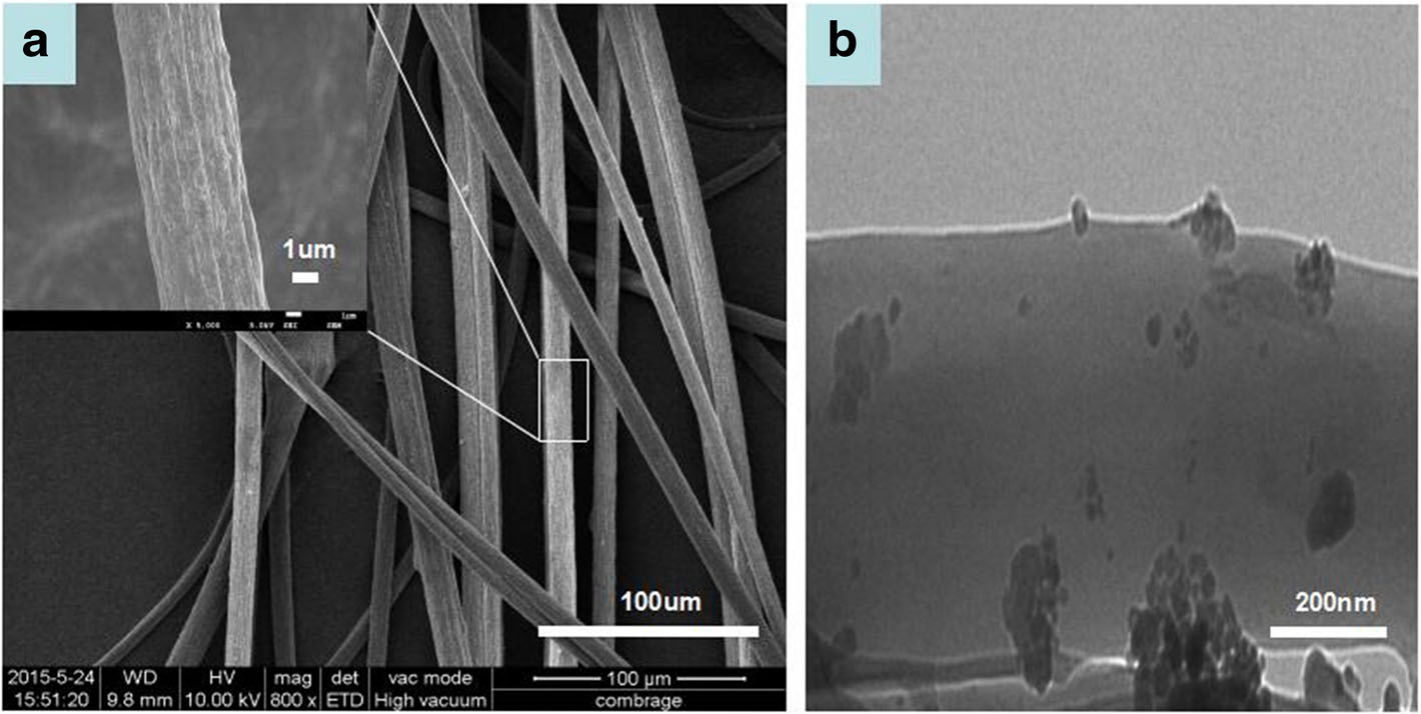
**a** SEM and **b** TEM images of the PVDF/γ-Fe_2_O_3_ microfibers

Figure 3a shows the SEM image of the PVDF/γ-Fe_2_O_3_ fibers. The average diameter of the fibers was confirmed as 8.4 μm. PVDF/γ-Fe_2_O_3_ fibers are drawn with solvent evaporated rapidly, so all fibers exhibited rough surfaces. More details about the inner structure of the fiber are shown in the TEM image (Fig. 3b). It is apparent that most γ-Fe_2_O_3_ nanoparticles are agglomerating because it is difficult to disperse the nanoparticles uniformly in the spinning solution with high viscosity. The average diameter of the dispersed nanoparticles is about 20 nm.

### Infrared Spectroscopy of PVDF Powder and PVDF/γ-Fe_2_O_3_ Fibers

**Fig. 4:**
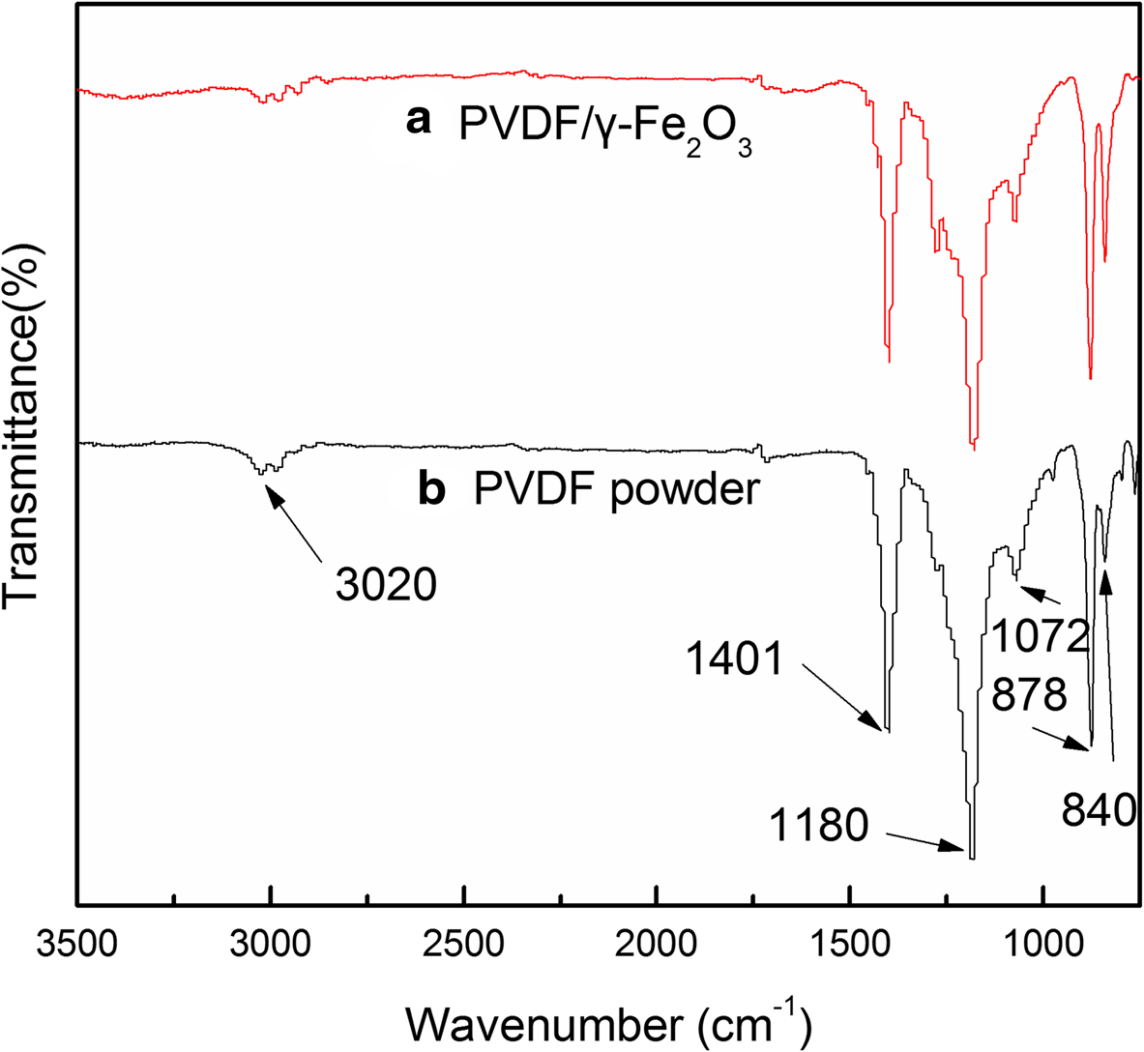
FTIR spectra of (**a**) as-spun PVDF/γ-Fe_2_O_3 _fibers and (**b**) PVDF powder

PVDF is a semi-crystalline polymer and its structure is long molecule chain, which consists of repeated unit CH_2_CF_2_ [22]. Infrared spectroscopy was applied to characterize the structure of the PVDF powder and the resulting PVDF/γ-Fe_2_O_3_ fibers. Fig. 4 shows the FTIR spectra of the composite fibers and PVDF powder. The bands appearing at 3020 and 1401 cm^−1^ are assigned to C–H stretching and C–H deformation, respectively [23]. Band at 1180 cm^−1^ is assigned to C–F stretching [24]. Moreover, absorption bands at 1072, 878, and 840 cm^−1^ are indications of β-crystal of PVDF [25–27]. The wavenumbers of the FTIR peaks and their assignments of different groups and crystallites are exhibited in Table 2. Here, it is noted that the characteristic peaks of γ-Fe_2_O_3_ did not appear in the infrared spectra of the PVDF/γ-Fe_2_O_3_ fibers. So, the FTIR spectra of the composite fibers are quite similar to the PVDF powder.

**Table 2:** FTIR peak assignments for the PVDF/γ-Fe_2_O_3_ fibers

Wave numbers (cm^−1^)	Functional groups and crystallites
3020	C–H stretching
1401	C–H deformation
1180	C–F stretching
1072 878 840	β-crystal of PVDF

### The Relation Between Rotating Speed and Fiber Diameter

**Fig. 5:**
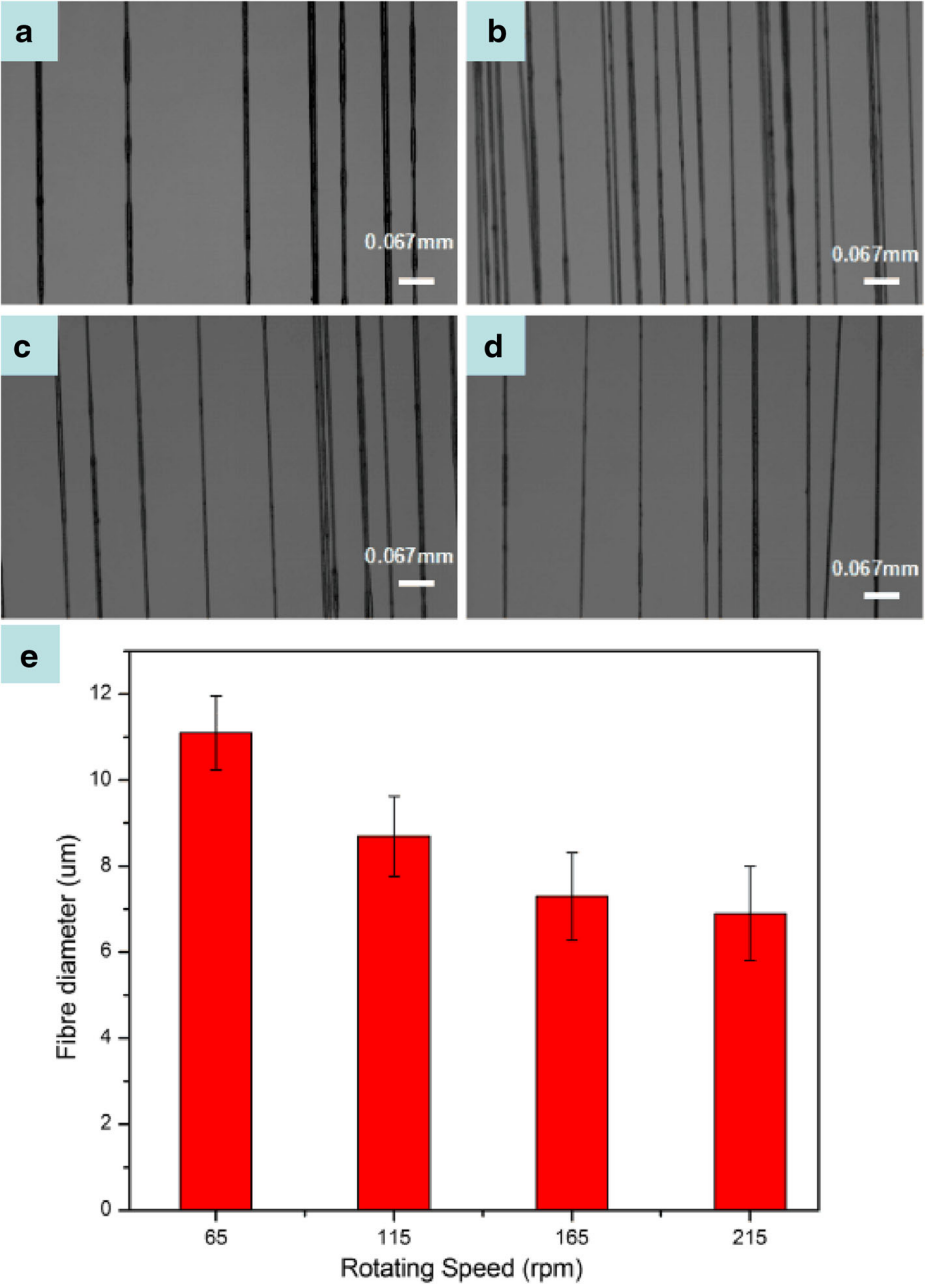
Optical photograph of fibers under different rotating speeds: **a** 65, **b** 110, **c** 175, and **d** 210 rpm. **e** Diagram of fiber diameter and rotating speed

In this method, fiber diameter can be changed by adjusting the rotating speed of the collecting stage. The relationship between rotating speed and fiber diameter was also studied. The distance between magnet and the tip of needle was insured as 7 mm. The rotating speed was set at 65, 115, 165, and 215 rpm (revolutions per minute), respectively. Fibers produced under different rotating speed are shown in Fig. 5. It can be seen obviously that fiber tapers with the increasing rotating speed. But when the rotating speed is beyond 215 rpm, fiber breakage frequency will increase during magneto-mechanical drawing process. When the rotating speed is too quick, the droplet transition from the needle to the magnet cannot succeed. The droplet is not able to overcome the surface tension and cannot attach to the magnet.

### Preparation of Well-organized Fibers

**Fig. 6:**
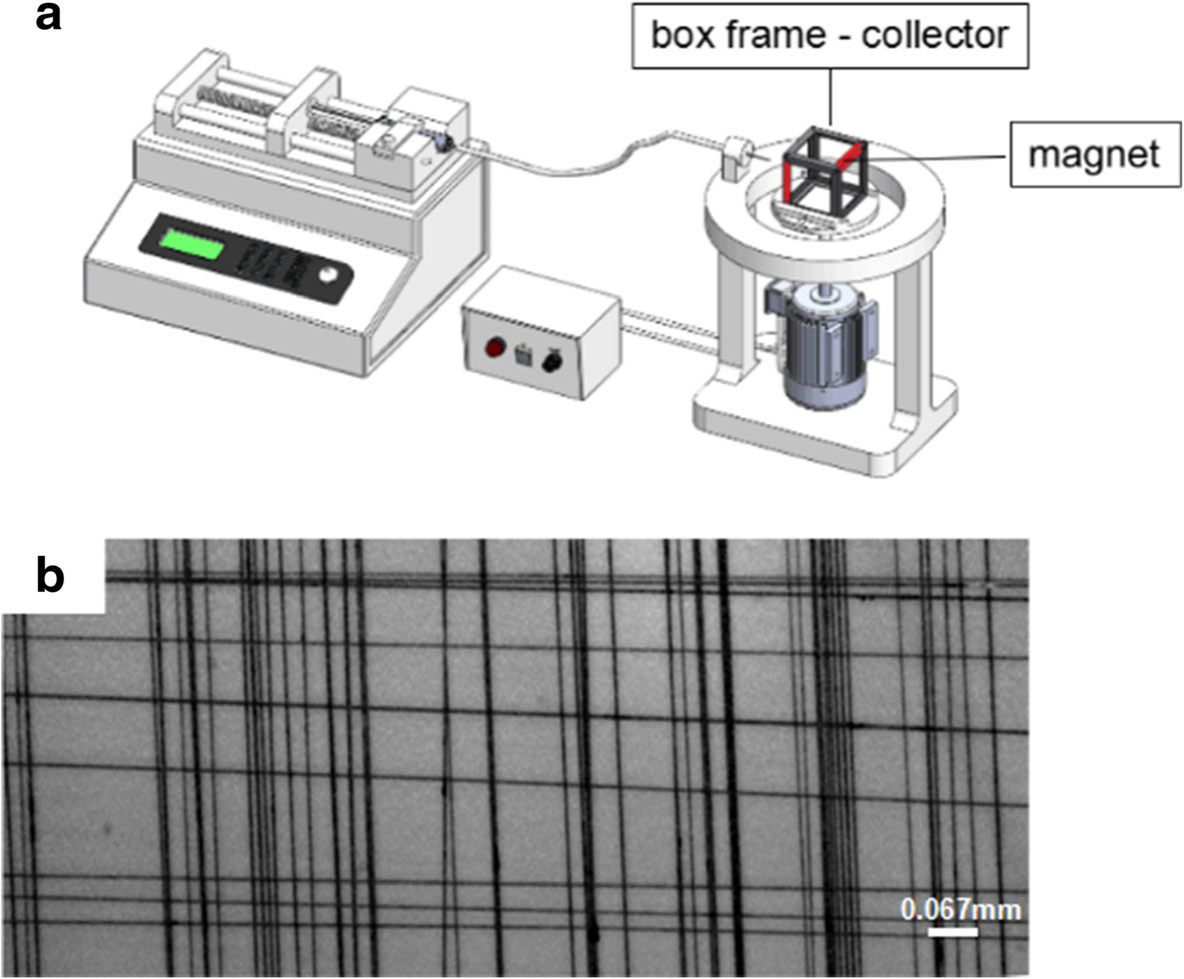
**a** A schematic diagram of improved device that can prepare vertical crossed fibers. **b** Optical photograph of vertical crossed fibers

Because of magneto-mechanical drawing mechanism and set-up, we can easily produce fibers in parallel. Fibers with vertical cross structure can also be prepared by changing the collecting device. A schematic diagram of improved equipment for vertical cross fibers is well presented in Fig. 6a, and the rotating stage was replaced by a removable box frame-collector. The red and gray colors of the box represent, respectively, the magnet and pillar. Firstly, fibers were collected on the frame via a few minutes of magneto-mechanical drawing. Then rotate the frame by 90°. And magneto-mechanical drawing continued for few minutes, resulting in vertical crossed fibers. This is a fast and practical method to fabricate crossed fibers or devices. Figure 6b shows the optical photograph of vertical crossed fibers.

### Magneto-Mechanical Drawing Other Composite Fibers

**Fig. 7:**
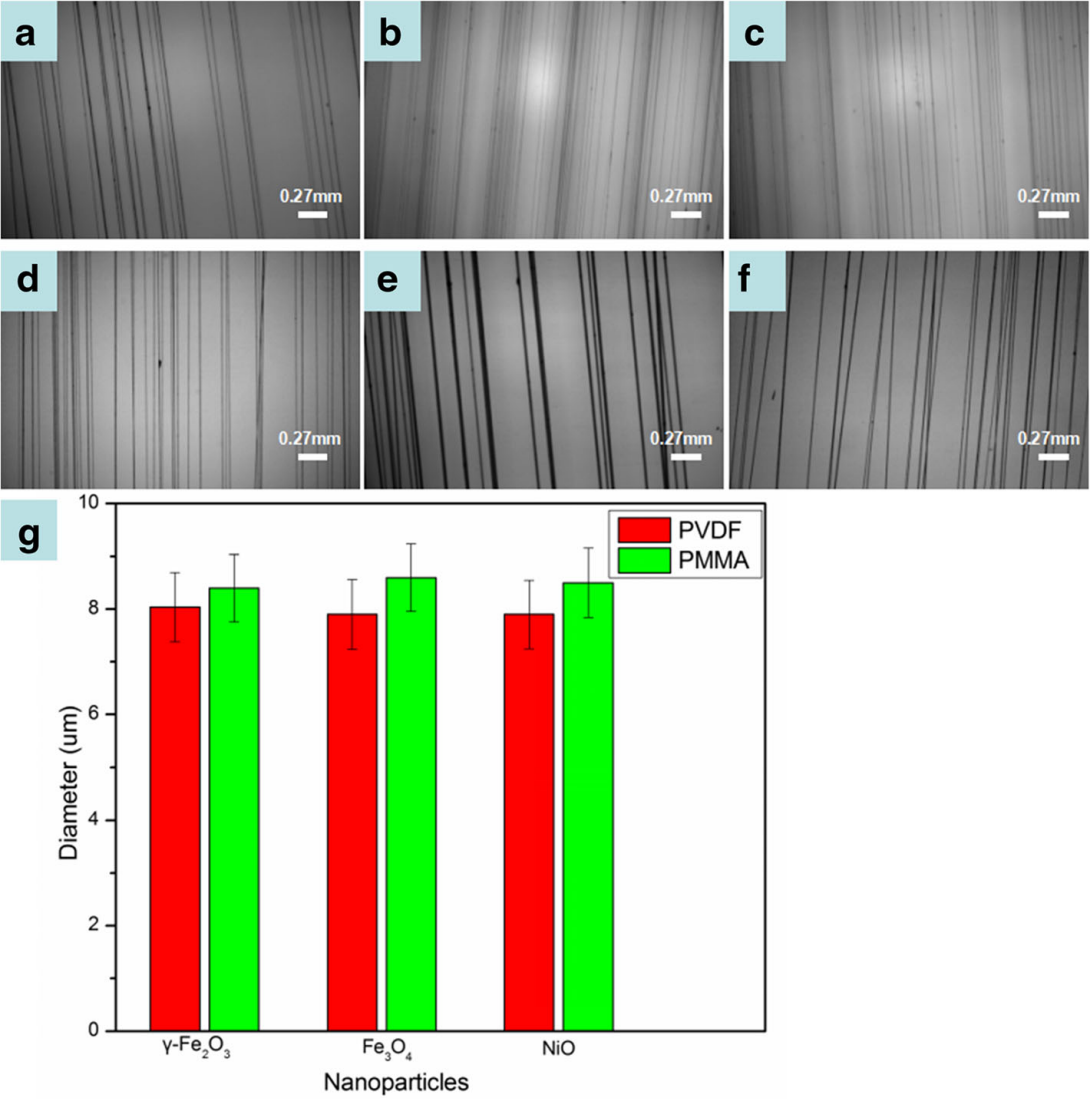
Optical photographs of composite fibers: **a** PVDF/γ-Fe_2_O_3_, **b** PVDF/Fe_3_O_4_, **c** PVDF/NiO, **d** PMMA/γ-Fe_2_O_3_, **e** PMMA/Fe_3_O_4_, **f** PMMA/NiO, and **g** the histogram of fiber diameter, polymer, and nanoparticle

Moreover, we use different polymers in this study to ensure the general applicability of magneto-mechanical drawing, including PVDF and PMMA. Three kinds of magnetic nanoparticles, including γ-Fe_2_O_3_, Fe_3_O_4_, and NiO, are also used. Similar to the process of preparing PVDF solution, 22 wt.% PMMA in DMF with 2 wt.% γ-Fe_2_O_3_ nanoparticles can be obtained. Besides, γ-Fe_2_O_3_ can be replaced by other magnetic nanoparticles. Additional file 1: Figure S2 shows the viscosity versus shear rate curves for different PMMA/magnetic nanoparticle solutions. As shown in Fig. 7, composite fibers of different materials were successfully produced by magneto-mechanical drawing. Figure 7g shows the effect of different polymer and magnetic nanoparticles on fiber diameter. The average diameter of PMMA/nanoparticle microfibers is larger than that of PVDF/nanoparticle fibers. Different types of magnetic nanoparticles have little influence on the average fiber diameter.

### Mechanical Property of PVDF/γ-Fe_2_O_3_ Fibers

**Fig. 8:**
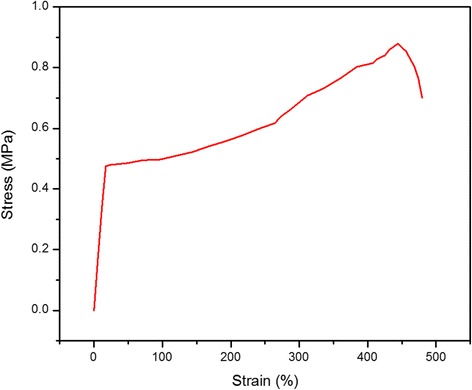
Stress-strain plot of the PVDF/γ-Fe_2_O_3_ fiber bundle

In order to find the potential application of fibers in stretchable component, mechanical deformation of PVDF/γ-Fe_2_O_3_ fibers was investigated by the stress-strain curve. A bunch of fibers (average bundle diameter ~10 μm) was stretched under increasing strain in longitudinal direction. The curve in Fig. 8 shows the mechanical properties of fibers: yield stress of 0.51 MPa, tensile strength of 0.88 MPa, yield strain of 24 % with an elongation at break of 444 %. Besides, three regions were exhibited in this curve. The elastic region is about 0 to 24 %, the plastic region is from 24 to 444 %, and the fracture region is from 444 to 483 %.

### Magnetic Property of the Fibers

**Fig. 9:**
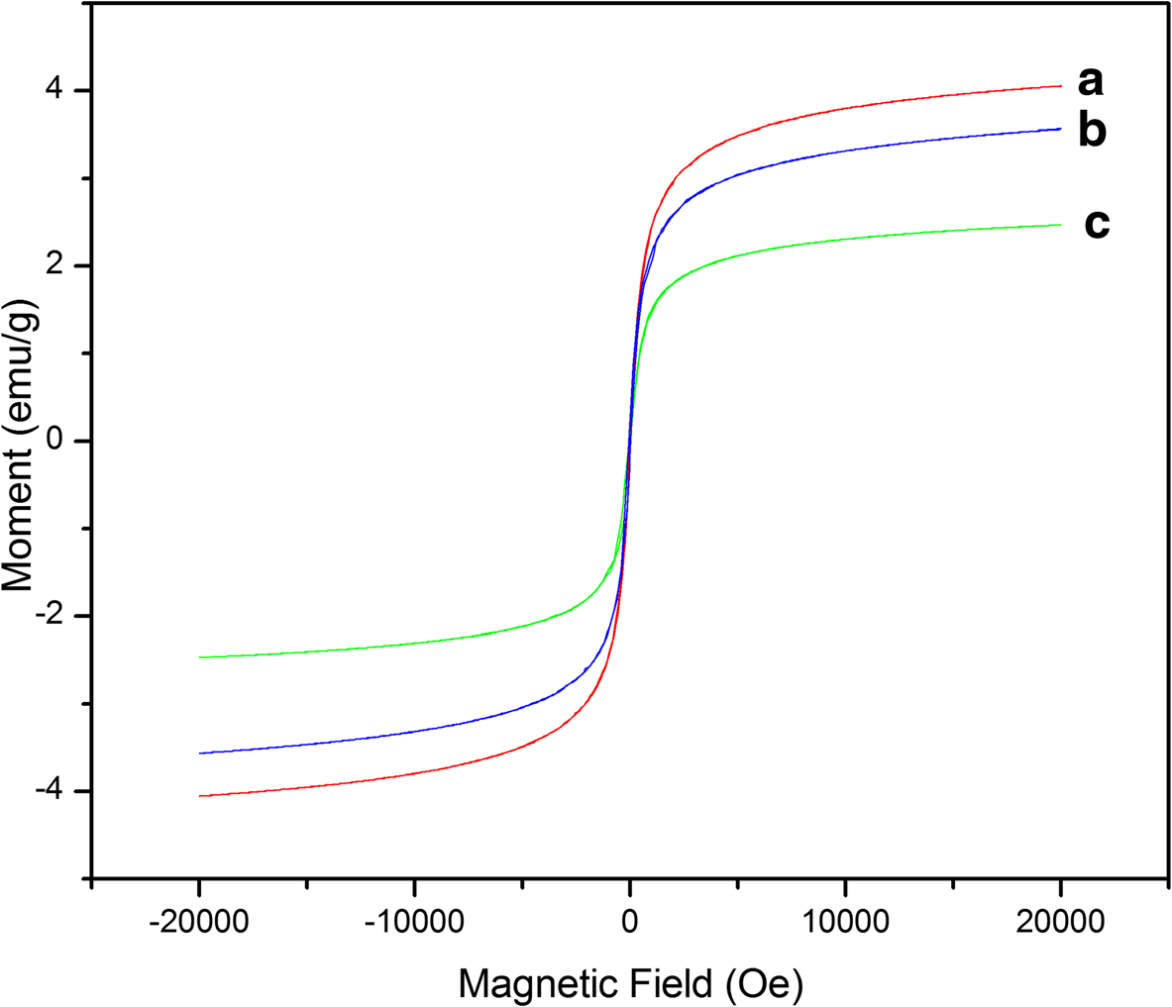
Magnetic hysteresis loops of the PVDF fibers with different magnetic nanoparticles: **a** Fe_3_O_4_, **b** NiO, and c γ-Fe_2_O_3_

Figure 9 and Additional file 1: Figure S3 show the magnetization curves of PVDF/nanoparticles microfibers and PMMA/nanoparticles microfibers at room temperature, respectively. Each of the magnetization curves shows a very small hysteresis loop that is hardly observed. The coercive force and remnant magnetization are almost zero, which indicates the superparamagnetic behavior of the fibers. The superparamagnetic phenomenon is due to the small size of magnetic nanoparticles (spherical single particle with diameter of 20 nm) inside the fibers [28, 29]. However, it should be noted that the coercive force does not decrease to zero, which could be attributed to the aggregated nanoparticles, as shown in Fig. 3b. The magnetic property may demonstrate potential applications of the composite fibers in magnetic sensors and drug delivery.

## Conclusions

In this study, a facile magneto-mechanical drawing method has been used to prepare magnetic composite microfibers. This method utilizes magnetic force generated by a revolving permanent magnet to draw polymer/magnetic nanoparticle solutions, which is simple, energy-saving, and safe. PVDF/γ-Fe_2_O_3_ fibers with parallel and crossed structures were successfully prepared by this method. SEM and TEM images indicate that the average fiber diameter is 8.4 μm and magnetic γ-Fe_2_O_3_ nanoparticles are distributed in the PVDF matrix. In addition, the fiber diameter decreases gradually by increasing rotating speed of collecting stage. Different polymers and magnetic nanoparticles have also been applied in this work successfully to prove the general applicability of the method. Particularly, the resultant fibers show excellent superparamagnetic behavior and ultra-high stretchability (~440 %), indicating potential applications in functional fibers, stretchable devices/sensors, and magnetic drug delivery.

## Additional file

Additional file 1: Figure S1. Viscosity versus shear rate curves for different PVDF/magnetic nanoparticle solutions (22 wt.% PVDF, 2 wt.% nanoparticles): (a) PVDF, (b) PVDF/γ-Fe_2_O_3_, (c) PVDF/Fe_3_O_4_, and (d) PVDF/NiO. **Figure S2.** Viscosity versus shear rate curves for different PMMA/magnetic nanoparticle solutions (22 wt.% PMMA, 2 wt.% nanoparticles): (a) PMMA/γ-Fe_2_O_3_, (b) PMMA/Fe_3_O_4_, (c) PMMA, and (d) PMMA/NiO. **Figure S3.** Magnetic hysteresis loops of PMMA composite fibers with different magnetic nanoparticles: (a) PMMA/Fe_3_O_4_, (b) PMMA/NiO, and (c) PMMA/γ-Fe_2_O_3_. (DOC 306 kb)
