# Supplementary material for: Fabrication of Continuous Microfibers Containing Magnetic Nanoparticles by a Facile Magneto-Mechanical Drawing
Source: Nanoscale Res Lett. 2016 Sep 23;11:426. doi: 10.1186/s11671-016-1646-8 (PMC5035288; doi:10.1186/s11671-016-1646-8)
Supplement: Additional file 1: Figure S1. — Viscosity versus shear rate curves for different PVDF/magnetic nanoparticle solutions (22 wt.% PVDF, 2 wt.% nanoparticles): (a) PVDF, (b) PVDF/γ-Fe2O3, (c) PVDF/Fe3O4, and (d) PVDF/NiO. Figure S2. Viscosity versus shear rate curves for different PMMA/magnetic nanoparticle solutions (22 wt.% PMMA, 2 wt.% nanoparticles): (a) PMMA/γ-Fe2O3, (b) PMMA/Fe3O4, (c) PMMA, and (d) PMMA/NiO. Figure S3. Magnetic hysteresis loops of PMMA composite fibers with different magnetic nanoparticles: (a) PMMA/Fe3O4, (b) PMMA/NiO, and (c) PMMA/γ-Fe2O3. (DOC 306 kb) [file 11671_2016_1646_MOESM1_ESM.doc]

**Fabrication of continuous microfibers containing magnetic nanoparticles by a facile magneto-mechanical drawing**

Jin-Tao Li ^a) †^, Xian-Sheng Jia ^a) †^, Gui-Feng Yu ^a) †^, Xu Yan ^a), b)^, Xiao-Xiao He ^a)^,

Miao Yu ^a), c)^, Mao-Gang Gong ^a)^, Xin Ning ^b), d)^, and Yun-Ze Long ^a), b) *^

**Supporting Information**

**
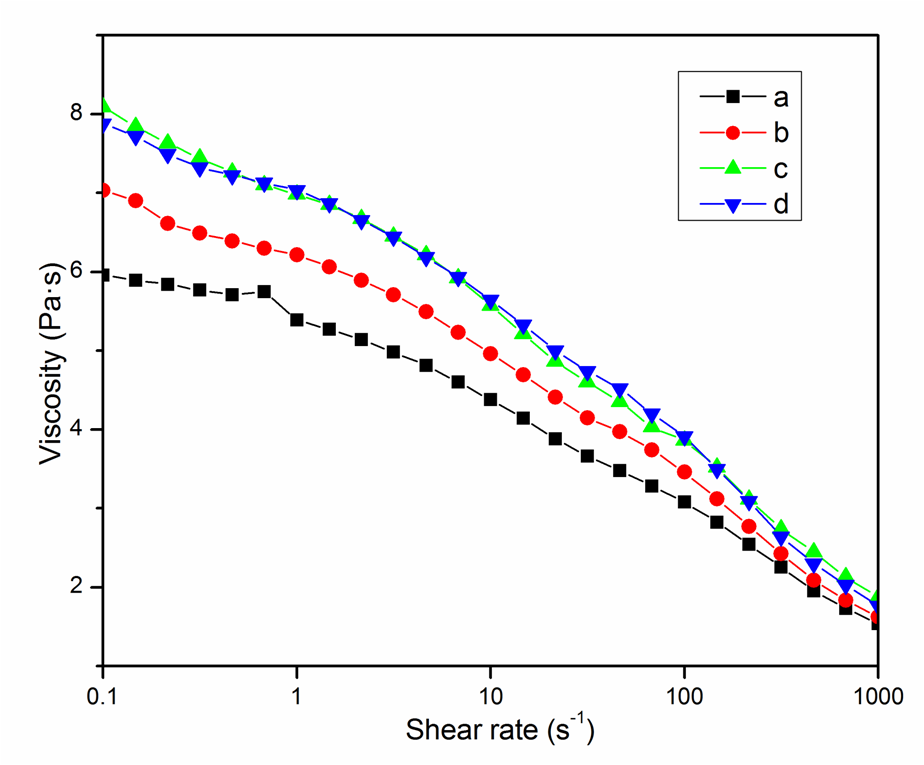
**

**Fig. S1** Viscosity versus shear rate curves for different PVDF/magnetic nanoparticle solutions(22wt% PVDF, 2wt% nanoparticles): (a) PVDF, (b) PVDF/γ-Fe_2_O_3_, (c) PVDF/Fe_3_O_4_, and (d) PVDF/NiO.


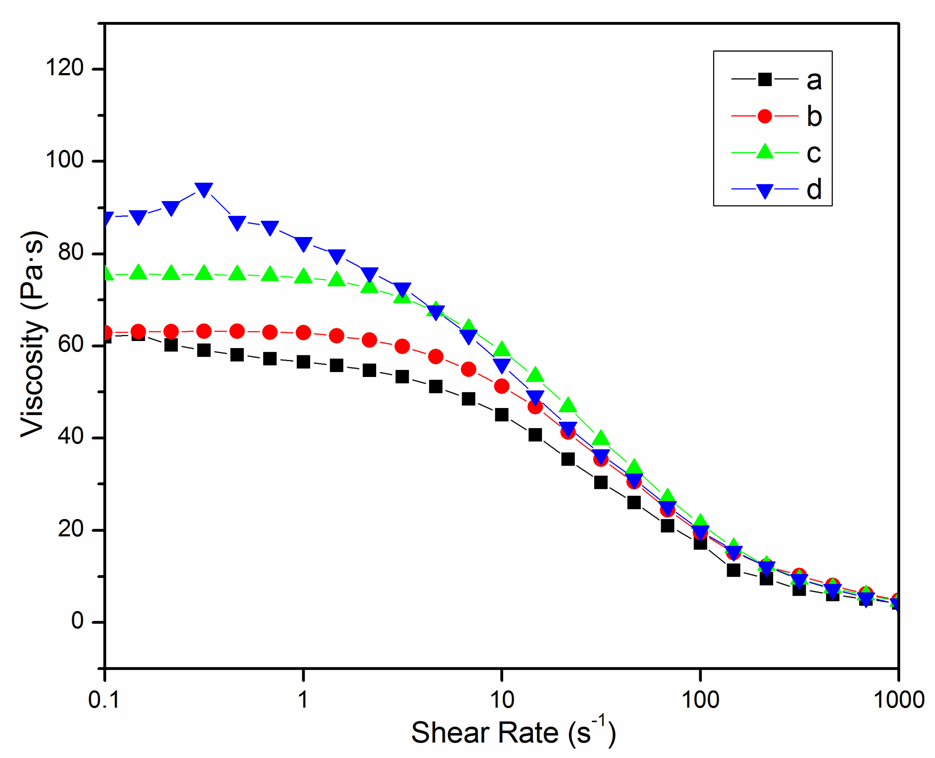


**Fig. S2** Viscosity versus shear rate curves for different PMMA/magnetic nanoparticle solutions(22wt% PMMA, 2wt% nanoparticles): (a) PMMA/γ-Fe_2_O_3_, (b) PMMA/Fe_3_O_4_, (c) PMMA, and (d) PMMA/NiO.


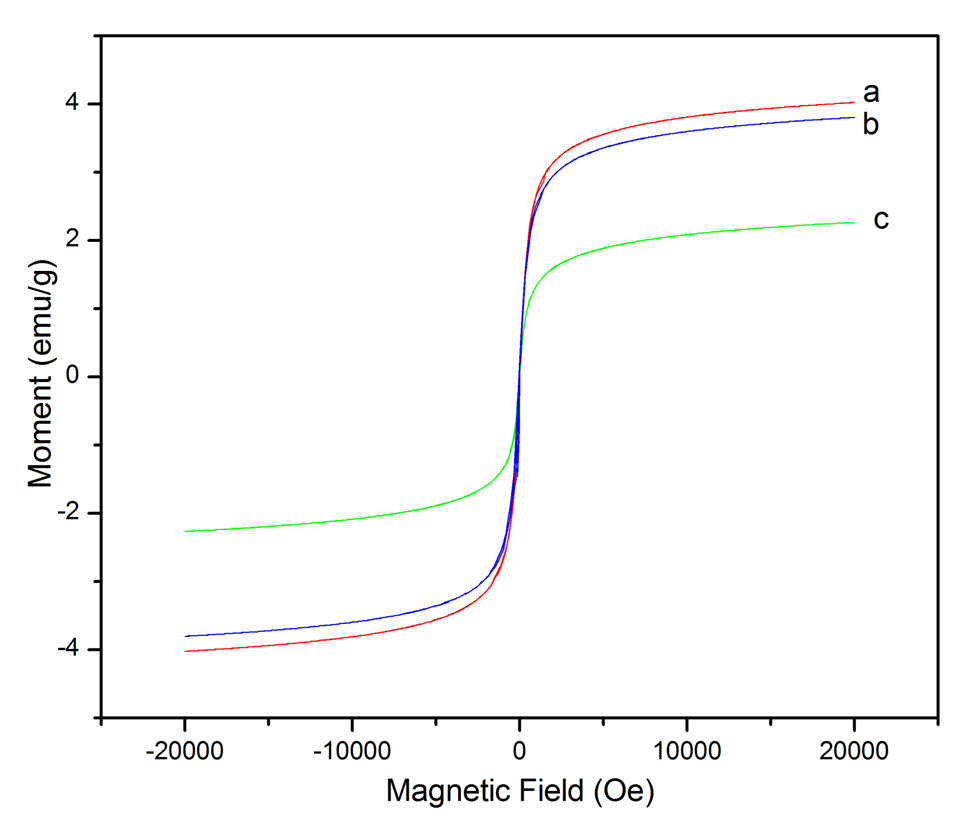


**Fig. S3** Magnetic hysteresis loops of PMMA composite fibers with different magnetic nanoparticles: (a) PMMA/Fe_3_O_4_, (b) PMMA/NiO, and (c) PMMA/γ-Fe_2_O_3_.
